# Single fathers’ experiences of using egg donation and surrogacy to start a family

**DOI:** 10.1093/humrep/dead152

**Published:** 2023-08-01

**Authors:** C Jones, V Jadva, S Zadeh, S Golombok

**Affiliations:** Social, Genetic and Developmental Psychiatry Centre, King’s College London, London, UK; Centre for Family Research, University of Cambridge, Cambridge, UK; UCL Institute for Women’s Health, University College London, London, UK; Thomas Coram Research Unit, University College London, London, UK; Centre for Family Research, University of Cambridge, Cambridge, UK

**Keywords:** surrogacy, egg donation, single fathers, experiences, qualitative research

## Abstract

**STUDY QUESTION:**

What are the experiences of single men using egg donation and surrogacy as a route to parenthood?

**SUMMARY ANSWER:**

The fathers mainly had a positive relationship with the surrogate and simultaneously exercised agency, and experienced challenges, during the process of surrogacy.

**WHAT IS KNOWN ALREADY:**

Little is known about single men’s experiences of egg donation and surrogacy arrangements. Studies have focused on single men’s decision-making processes about the use of surrogacy and family functioning once these families are formed. Questions remain about how fathers experience and navigate the process of surrogacy as a single man.

**STUDY DESIGN, SIZE, DURATION:**

The study is an international, in-depth qualitative study of fathers who chose to begin a family and parent alone. Data were collected between 2018 and 2021 as part of a larger study of solo fathers with different routes to parenthood. The present study reports on 21 fathers who used surrogacy and egg donation to begin their family. The average age of the fathers was 44 years, the fathers had young children aged 6 years or younger, and lived in countries across Australia, Europe, and North America.

**PARTICIPANTS/MATERIALS, SETTING, METHODS:**

Purposive sampling was used to recruit participants. In-depth semi-structured interviews were conducted. Interview topics included fathers’ experiences of the process of using egg donation and surrogacy, and navigating the relationship with the surrogate. The audio-recorded interviews lasted around 2 hours and were subsequently transcribed verbatim.

**MAIN RESULTS AND THE ROLE OF CHANCE:**

Data were analysed using reflexive thematic analysis and qualitative content analysis. Most of the fathers chose an identifiable egg donor. Regarding the relationship with the surrogate, many fathers had remained in contact with her, but to differing degrees, and they generally reported positive relationships. Thematic analysis led to the identification of three themes relating to the fathers’ experiences of choosing surrogacy as a single man: the ability to make choices; challenges and constraints; and special relationship.

**LIMITATIONS, REASONS FOR CAUTION:**

Due to the variation between different countries regarding laws on surrogacy, contextual factors may have impacted on the experiences of single fathers, and the sample size was small. However, the research provides new insights into an area with little academic literature.

**WIDER IMPLICATIONS OF THE FINDINGS:**

Given the growing trend of single men having children through surrogacy, the findings suggest that this new path to parenthood can be both rewarding and challenging. Single men may benefit from tailored support and counselling to help them navigate the surrogacy journey.

**STUDY FUNDING/COMPETING INTEREST(S):**

This study was funded by the Wellcome Trust (grant number 208013/Z/17/Z). The authors have no conflicts of interest to declare.

**TRIAL REGISTRATION NUMBER:**

n/a

## Introduction

The past few decades have seen an increase in family diversity, with the use of assisted reproduction to become a parent becoming increasingly popular. One form of assisted reproduction that has remained controversial is surrogacy. Surrogacy refers to the act of a person carrying a pregnancy on behalf of intended parent(s). Historically, surrogacy arrangements have most often been pursued by cisgender heterosexual couples. However, recent years have seen important changes to the law, with some countries giving access to surrogacy to same-gender male couples, and more recently, single men. Single men who use surrogacy to start a family can either use traditional surrogacy, whereby the surrogate’s own egg is used, or gestational surrogacy, where a separate donor’s egg is used. Intended parent(s) may choose an egg donor who is known to them. Alternatively, the egg donor may be identifiable or ‘identity-release’, where the child has the possibility of knowing the identity of their egg donor at a later age, or anonymous, dependent on the jurisdiction in which the surrogacy arrangement is conducted.

Access to surrogacy and laws on assisted reproduction vary significantly internationally. In much of Europe, for example, in Italy, Spain, France, and Germany, surrogacy is not legal. In the UK, legal changes in 2019 have enabled surrogacy to be a family-building option for single men (Department of Health and Social Care, 2021). At that time, single applicants were granted the ability to apply for a parental order to transfer legal parenthood from the surrogate to themselves, with the surrogate’s consent.

Given that access to surrogacy by single men remains restrictive in many countries, some fathers opt for international surrogacy. Countries also vary in how surrogacy is practiced, which can also influence intended parents’ reasons for travelling overseas (Jadva *et al.*, 2021). In some locations, such as the US, the surrogate is allowed to receive some financial compensation, although surrogacy and its costs are regulated on a state rather than national basis (Birenbaum-Carmeli and Montebruno, 2019), whereas in other locations, such as the UK, financial compensation is not allowed, other than costs directly associated with the pregnancy (Department of Health and Social Care, 2021). Where surrogates are located may also impact the types of relationships that intended parents form with their surrogate (Jadva *et al.*, 2019).

### Planned single parent families

Although research on single parent families has generally focused on parents who did not plan to raise a family alone, for example, single parents through divorce (Coles, 2015), increasing numbers of parents elect to begin their family alone. These families have been found to have different characteristics to other types of single parent families (Golombok, 2020), thus warranting research specific to these family types, their experiences of starting their family, and of family life.

Research on solo mothers, sometimes also termed single mothers by choice (Golombok *et al.*, 2016), has found that women choose to begin their family alone because they want to have a child and be a mother, and feel that time is running out for them because of their increasing age (Jadva *et al.*, 2009). Similar motivations among Italian single fathers were found by Carone *et al.* (2017), who reported that the fathers felt it was the right time in their life to become a parent, due, for example, to financial security or their age.

Despite these similarities between solo mothers and fathers, there may be experiences and challenges unique to becoming a single father. Solo fathers challenge the perception that women have a more active desire to become a parent than men (Johnson, 2017). Despite a wealth of research evidence demonstrating that a two-parent, cisgender, heterosexual couple family is by no means necessary for optimal child adjustment (Golombok, 2015; Imrie and Golombok, 2020), ‘traditional’ family forms remain privileged (Johnson, 2017); representations of fathers in the media perpetuate stereotypes and do not account for the variety of different family types or the diversity of fathers’ experiences (Wall and Arnold, 2007; Gregory and Milner, 2011). As such, it is possible that single men will face specific challenges in pursuing parenthood alone due to these lasting assumptions, alongside facing practical and legal barriers to parenthood.

### Families through surrogacy

Regarding the choice of surrogacy as a route to parenthood, Carone *et al.* (2017) found that heterosexual single fathers in Italy had considered, or had had, sexual intercourse with women to become a father. Most gay solo fathers reported that they had always wanted to use surrogacy; the adoption rules in Italy prevent single men and women from adopting children and most fathers stated that having a genetic connection to their child was an important reason for pursuing surrogacy (Carone *et al.*, 2017).

Research addressing fathers’ experiences of surrogacy has been limited and has focused on fathers in couples (Imrie and Golombok, 2020), with experiences differing by family type. For example, in a study of surrogates where the intended parents were heterosexual couples, fathers were less involved in the process of surrogacy than were mothers (Jadva *et al.*, 2003). Carone *et al.* (2018) found fathers in same-gender couples using overseas surrogacy to be actively engaged in the pregnancy process, despite the physical distance between them and their surrogate, and they remained in frequent communication with her. In contrast, fathers reported a more distant relationship with the egg donor (Carone *et al.*, 2018), a finding similar to a US study of coupled gay fathers (Blake *et al.*, 2016). The fathers in this latter study were mostly happy with how much contact they had with the surrogate, though a few wanted to have more contact. Fathers with international surrogacy arrangements have reported facing difficulties in feeling involved in the pregnancy, and in navigating the legal framework, particularly regarding registering parentage on the child’s birth certificate (Carone *et al.*, 2021b). Guidelines and procedures vary between countries, adding an additional layer of complexity.

In terms of fathers’ wider experiences of the process, many of the fathers in Blake *et al.*’s (2016) US study felt supported by their family and friends in their use of surrogacy and were satisfied with their surrogacy experience. However, research in Israel has shown that whilst coupled gay fathers through surrogacy report greater life satisfaction than do heterosexual fathers, they also report elevated levels of postnatal depression (Shenkman *et al.*, 2022). By way of explaining these findings, the authors suggested that fathers might experience cumulative stress, that is, the stress experienced by gay fathers as a minority group in addition to the common strain of the transition to parenthood.

In terms of parent and child adjustment in two-father families formed by surrogacy, the US study found that the children, aged around 5 years on average, showed no differences in behavioural difficulties, and lower levels of emotional difficulties, than children in two-mother families formed through sperm donation (Golombok *et al.*, 2018). Similarly, the Italian study found no differences in psychological adjustment between children in single father and two-father families formed through surrogacy (Carone *et al.*, 2020, 2021a).

Research on fathers’ experiences of using egg donation and surrogacy in same-gender couples sheds light on how those in father-headed families might navigate, and think and feel about, the process. However, there is a paucity of research on single fathers’ experiences of surrogacy arrangements, both during and after the surrogacy takes place. From an intersectional perspective (Collins, 1998; Bowleg, 2008), the fathers’ relationship status as single, their male gender identity, their socio-economic status (especially considering the high costs often encountered with surrogacy), and other aspects of their identity, such as their sexual orientation, may lead to a unique surrogacy experience different to that experienced by couples. The recently emerging body of work on single fathers through surrogacy indicates that these fathers often simultaneously experience social acceptance and discrimination (Jones *et al.*, 2022; Tsfati and Segal-Engelchin, 2022; Zadeh *et al.*, 2022). Yet, little is known about how single fathers navigate contact with all parties involved in the surrogacy and egg donation, and their thoughts and feelings about the surrogacy process.

The current study aimed to provide insights into single men’s experiences of egg donation and surrogacy to become parents, and how they negotiate a relationship with the surrogate before and after the birth of the child. Due to between-country variation in terms of access to surrogacy, rules and regulations, and compensation, the present paper focuses on the fathers’ experiences of their relationship with the surrogate and the surrogacy and egg donation process, rather than the nuances of arranging surrogacy in specific locations.

## Materials and methods

### Sample characteristics

The sample comprised 21 fathers with an average age of 44 years. The fathers were cisgender men and in terms of sexual orientation, 19 fathers were gay, one was heterosexual, and one was asexual. Thirteen fathers had one child and eight fathers had two children. The children were aged 0–6 years. At the time of starting the process of surrogacy, the fathers were single, but some later went on to start relationships. The sample was international, and among the 20 fathers (95%) who provided further demographic information, 15 identified as a European nationality, two as European and American, one as European and Asian, one as European and Australian, and one as Australian. Sixteen fathers described their ethnicity as white, three as mixed, and one as other (using the UK Office for National Statistics classification). The data form part of a larger study that included solo fathers through adoption (see Zadeh *et al.*, 2022). The fathers were recruited with support from Brilliant Beginnings, Cafcass, Circle Surrogacy, Growing Families and Family Equality, and through snowballing. The study received ethical approval from the University of Cambridge Psychology Research Ethics Committee.

All fathers in the present study used gestational surrogacy, whereby a separate donor’s egg was used, i.e. the surrogate did not use her egg for the pregnancy. The fathers underwent surrogacy in North America, South America, Europe, and Asia. The US was the most common location, with 13 of the fathers undergoing surrogacy there. All fathers expressed a strong desire to be a parent as their motivation to start their journey to parenthood alone, although the reasons for choosing to become a single parent at the time they elected to do so were varied (see Jones *et al.*, 2022, for more information).

### Interviews

Semi-structured interviews were conducted by three researchers trained in the study techniques, either in person (n = 5), or over Skype or telephone (n = 16), given the study’s international nature. Once participants had read the information sheet and had the opportunity to ask any questions, written informed consent was obtained. Each interview lasted ∼2 h. The interviews were audio-recorded and transcribed and all identifying information was removed from the transcripts. The interview schedule had two main sections, addressing: (i) parent–child relationships, children’s adjustment, and fathers’ well-being; and (ii) fathers’ route to parenthood, experiences of surrogacy, disclosure of surrogacy and egg donation, and experiences of being a single father. The interview was semi-structured, so each father was asked similar questions, but prompts were used to elicit more information from their responses or to elaborate on different topics. Considering the wide range of topics covered in the interview, only the second section of the interview was analysed, as this section covered the topics that were relevant for the focus of the present investigation.

### Analysis

The study addressed two research questions:

1. What are single fathers’ choices and preferences when choosing an egg donor?
2. How do single fathers describe their experience of surrogacy and their relationship with the surrogate?

### Choices and preferences when choosing an egg donor

Fathers’ answers to questions regarding the egg donor’s status (anonymous or identifiable) and their preferred status for the donor (anonymous, identifiable, or open to both) and whether they were in contact with the egg donor (in contact, no longer in contact, not in contact) were coded as above. Illustrative quotes are presented with the findings.

Subsequently, to analyse fathers’ responses about the characteristics they were looking for when searching for an egg donor, qualitative content analysis was used. Qualitative content analysis is a method for exploring the experiences and narratives of a sample by creating categories to describe participants’ responses, allowing for counts to be made of participants in each category. It applies the benefits of quantitative analysis to text-based data (Mayring, 2015). In the present study, it was chosen as a method to analyse the parts of the interview that were not open-ended (Neergaard *et al.*, 2009). The lead researcher (C.J.) read through all the responses and created data-driven categories. Illustrative quotes are presented alongside the table. Counts were made of each code and percentages presented.

### How do single fathers describe their experience of surrogacy and their relationship with the surrogate?

Regarding fathers’ experiences of their relationship with the surrogate, counts were made of the fathers’ answers to questions about current contact with the surrogate (in contact or not in contact) and frequency of current contact with the surrogate (in contact more than once a week, in contact once a week to once a month, in contact once a month to once every 3 months, in contact less than once every three months, and not at all in contact). Illustrative quotes are presented with the findings.

The section of the interview describing the father’s relationship with the surrogate was coded according to a coding scheme created for the purposes of the present study. The father’s relationship with the surrogate was rated as positive, neutral, ambivalent, or negative. The interviewer coded the father’s answers after the interview had finished and subsequently one-third of the interviews were rated by a second coder. Cohen’s Kappas were calculated to assess inter-rater reliability, Cohen’s Kappa = 0.72.

To enable a more in-depth understanding of the fathers’ experiences of the surrogacy process and the relationship with the surrogate, reflexive thematic analysis (Braun and Clarke, 2006, 2019, 2021) was used, with a particular focus on researcher positionality in the analytic process. Thematic analysis is about making sense of people’s experiences and understanding these as related to the social context (Braun and Clarke, 2019), hence is a useful methodological tool for analysing how the fathers’ status as a single man may have shaped some of their experiences of surrogacy. Line-by-line inductive coding of the transcripts was conducted; the codes were then grouped into similar codes and collapsed, and any codes that were not directly related to the research questions were excluded. The codes were then organized into themes and subthemes that were subsequently edited and refined by continually looking back at the transcripts and initial codes.

Regular peer debriefing enabled the researchers to reflect on the analysis throughout the process, which strengthened confidence in the findings (Flick, 2014). Also, the lead researcher (C.J.), who conducted the thematic analysis, continually reflected on her own positionality throughout the process of data collection, familiarization, and analysis. She considered her social context, and the aspects of her identity that may have shaped her understanding of the fathers’ narratives, to be critical and reflexive of the ways in which her meaning-making of the data might have been shaped by these influences (McCorkel and Myers, 2003). In particular, C.J. considered how as a female researcher who is not a parent, she would be mostly viewed as occupying an outsider status (Gair, 2012) regarding the fathers, and continually reflected on how this shaped her experience of conducting some, and analysing all, of the interviews.

## Results

### Research question one: Choices and preferences when choosing an egg donor

More of the fathers had used an identifiable egg donor (n* *=* *13, 62%) than an anonymous egg donor (n* *=* *8, 38%) (see Table 1). All fathers who used an identifiable egg donor reported that this was their preference. One father explained, ‘I’m a single gay man, it’s pretty obvious to my son that I didn’t make him on my own, so you know, I need to tell him and I want to be able to show him photographs and I want him to be able to have the option of getting in touch and seeing whether he wants to meet you when he’s grown up’. Of the fathers who had used an identifiable donor, some were in touch with the donor; four (31%) of the fathers were currently in contact, and a further two had been in contact since the birth of their child but not recently (identifiable also referred to donors whose identity had already been disclosed to the recipient. Rules regarding identifiability vary by country.)

**Table 1.** Types of donor and fathers’ preferences for donor identifiability by the type of donor they ended up using.

	Type of donor	
	Anonymous	Identifiable
	N (%)	N (%)
	8 (38%)	13 (62%)
**Preference**		
Anonymous	4 (50%)	0
Identifiable	2 (25%)	13 (100%)
Open to either anonymous or identifiable	2 (25%)	0
**Contact with the donor**		
In contact	N/A	4 (31%)
No longer in contact	N/A	2 (15%)
Not in contact	N/A	7 (54%)

Of the eight fathers who had used an anonymous donor, half said that had been their preference. Two explained that this had not been their preference; instead, an identifiable donor would have been their choice, with one father explaining, ‘I would have preferred that. I don’t even know if I can find out. I mean if they’re really interested, I guess we can hire a detective to try and … find her’. A further two fathers who had used an anonymous donor had been open to the idea of using an identifiable donor, but did not ultimately do so.

Regarding the characteristics the fathers were looking for when searching for an egg donor, a total of 10 criteria were mentioned across the sample (these are listed in Table 2). Only one father reported that he was not searching for particular qualities. For the remaining 20 fathers, the most cited commonly sought-after characteristics were good health (43%), general appearance (43%), and education level (38%). Fathers often described how the egg donor’s health was their primary concern: ‘The important thing to me was that she’s healthy, that she’s done it [surrogacy] before successfully’. However, fathers also discussed considering the egg donor’s appearance, either generally or specifically (e.g. in terms of, for example, eye colour, hair colour, and height). Some of the fathers reported that having a similar ethnic background was important to them: ‘I think just the ethnicity was a big thing because I wanted us to have the same skin colour’. A few of the fathers with identifiable donors had been able to talk to, or sometimes meet, the egg donor, prior to treatment. These fathers mentioned the importance of getting on well with the donor or of finding common ground: ‘I just felt very comfortable with the profile and the outlook in life and the description and the picture, and then the discussion more importantly on the phone’.

**Table 2.** Donor characteristics the fathers were searching for.

Characteristic	**N (%)** ^1^
Appearance (overall)	9 (43%)
Appearance (specific characteristics, e.g. eye colour, hair colour, height)	3 (14%)
Character	3 (14%)
Connection	1 (5%)
Education	8 (38%)
Ethnicity	6 (29%)
Family background	1 (5%)
Has children	1 (5%)
Health	9 (43%)
Interests	1 (5%)

1 The percentages add up to over 100% as many of the fathers mentioned they were searching for more than one characteristic.

### Research question two: How do single fathers describe their experience of surrogacy and their relationship with the surrogate?

#### Contact and relationship with the surrogate

Most (n =* *16, 76%) of the fathers were still in contact with the surrogate and many (n* *=* *14, 67%) had a positive relationship with her (see Table 3). Almost half of the fathers were in contact with the surrogate at least once a month (n* *=* *9, 43%).

**Table 3.** Fathers’ contact with the surrogate and relationship quality.

	N (%)	Illustrative quote
**Current contact with surrogate**		
Yes	16 (76%)	‘We still feel that we want to share how we are and I want to share how (child) is progressing.’
No	5 (24%)	‘We haven’t been in touch.’
**Frequency of contact with surrogate**		
More than once a week	4 (19%)	‘We’re in touch all the time … .Whatsapping, messaging to each other at least once per week.’
Once a week to once a month	5 (24%)	‘We’ll text and almost … like maybe once a week and we probably have a Facetime call once every month, every couple of months.’
Once a month to once every 3 months	4 (19%)	‘Half a dozen times this year, and that’s been exchanging photos, you know, doing a little thing for Mother’s Day.’
Less than once in 3 months	3 (14%)	‘Once. Basically on their birthdays.’
Not at all	5 (24%)	‘No, nothing at all.’
**Relationship with surrogate**		
Positive	14 (67%)	‘Amazing, I love her, seriously.’
Neutral	4 (19%)	‘It’s like a cousin, I’m going to see her from time to time, but it’s … and we have a good relationship, but we wouldn’t be friends probably.’
Ambivalent	3 (14%)	‘It wasn’t the best of relationships, it also wasn’t the worst.’
Negative	0	

#### Thematic analysis of fathers’ narratives of their experience of surrogacy and relationship with the surrogate

Findings centred around three main themes and twelve subthemes. The first theme ‘ability to make choices’ encompasses fathers’ experiences of (i) surrogacy as their preferred route to parenthood (ii) gathering knowledge, resources, and support, (iii) practicalities, and (iv) perceived importance of being genetically related. The second theme stands both alongside and in contrast to the first theme; ‘challenges and constraints’. This theme explains some of the difficulties the fathers faced, including (i) external circumstances, (ii) gatekeepers, (iii) location, and (iv) complexities in the relationship with the surrogate. The third theme ‘special relationship’ explores fathers’ feelings about the surrogate and their navigation of the relationship. Their narratives involved (i) gratitude and amazement, (ii) helping to feel involved, (iii) friendship, and (iv) seeing the surrogate as family-like. Altogether, these themes signify the journey fathers went through; the motivation to use surrogacy, the process leading up to the pregnancy, and how the fathers’ navigated the relationship with the surrogate during and after the pregnancy.

##### Theme one: Ability to make choices

This theme describes the resources the fathers had, and the decisions they were able to make, in their journey to parenthood.

###### Preferred route to parenthood

Fathers described choosing surrogacy over adoption partially because of a desire to have a genetic connection with their child and to be involved at the birth. One father stressed that he ‘wanted to go through the kind of childbirth as much as you can’. However, the fathers also discussed how surrogacy was viewed as a more favourable route to parenthood due to hesitancies over adopting as a single man, partly due to feeling discriminated against, and partly due to the lack of support from a partner. One father recalled: ‘when I went to look into this adoption … so firstly I wanted … firstly in adoption if you’re husband and wife you get top priority, then if you’re a lesbian couple, then if you’re a gay couple, then a single woman and then a single man, so for a start I was already at the bottom of the hierarchy’. Another father described his worries about whether he would be able to become an adoptive father as ‘the process can take years, it’s very difficult to get’ so he felt that by choosing surrogacy he was ‘more in control of the outcome’.

###### Gathering knowledge, resources, and support

When describing their preparations, many of the fathers referred to the research they had done, the people they connected to, and the support groups they had joined, each of which helped them to feel informed about the process and confident in their choices: ‘A lot of the people that I surrounded myself with had been through this process, so I always had someone to talk to, someone to discuss things with if I had any concern about anything’. Groups or forums were often seen as a useful, and easy, way to access help in the lead up to choosing surrogacy. Many of the fathers highlighted that they had sought out opportunities to feel more knowledgeable about surrogacy: ‘the way I managed to get information on how to do the surrogacy was through a support group … it’s more like a forum, like an association of lesbian and gay parents’. Fathers also referred to the physical resources, including their finances, that had helped them to prepare for choosing surrogacy: ‘it was possible, it was like, you know, I’m in the financial situation where I could do it’.

###### Practicalities

Fathers also outlined the decisions they had made regarding the practicalities of surrogacy, such as where to conduct the surrogacy arrangement. One father described how in choosing where to have surrogacy, he took different factors into account: ‘I knew that the medical care was really good, the surrogates were properly screened’. Fathers involved in transnational surrogacy arrangements described some anxiety about being far from the surrogate during the pregnancy, and their choices in navigating this. One father explained that he had carefully considered who he thought would be the right surrogate to carry the pregnancy: ‘Someone I could trust, especially knowing that most of the pregnancy would be spent far away’. Another father managed the physical distance by choosing a surrogate who lived near to his family, to give him ‘peace of mind’ if ‘anything happened during the pregnancy’.

Some of the fathers explained that they appreciated the role clinics and agencies took in managing the practicalities of surrogacy. One father explained: ‘part of the work of the agency is to take care of all the practical, legal, financial stuff so that you can focus on the human relationship’. Another father felt the agency had been particularly careful when matching him to a surrogate, making sure to reflect his wishes: ‘They [the agency] have way, way, way less surrogates than they have donors, but their choice was excellent, theirs was like spot on’.

###### Perceived importance of being genetically related

Many of the fathers valued having a genetic connection to their child. This was particularly evident when fathers discussed their decision-making about which route to parenthood they pursued. One father explained: ‘I just wanted him—or her, at that stage I didn’t know—to be mine’. Similarly, another father explained ‘I like the idea of having a baby of my own’. Notions of a genetic connection equating to parenthood were also present in the fathers’ narratives when discussing a desire to use a separate egg donor rather than the surrogate’s egg. This fed into the decisions and choices they made at the beginning of the surrogacy arrangement, motivated, in part, by wanting to protect their relationship with their future child. When talking through his initial thoughts at the time a surrogate had agreed to start a surrogacy arrangement, one father recalled: ‘I was thinking well you’re just growing my baby rather than giving me yours, yeah, so … I just felt more comfortable with it really. And, also, I was concerned about the bond and … things like that’. Similarly, when describing why he used gestational surrogacy, another father explained: ‘I mean I think it is sensible, there are reasons for it, it’s very clear that the surrogate is hosting this embryo, a baby, and it’s not hers, it’s never going to be hers, she’s giving it back’. The perceived advantages of using a separate egg donor and surrogate featured in many of the fathers’ narratives, highlighting the importance of a genetic connection for the fathers and their feelings about what the absence of a genetic connection between the surrogate and child represented.

##### Theme two: Challenges and constraints

Whilst many fathers described their ability to have control over the process of surrogacy and make choices and decisions, the fathers’ accounts also highlighted the complexities of the process, which involved many factors and different parties. Fathers explained that they sometimes lacked agency over aspects of the surrogacy.

###### External circumstances

Some of the fathers described the legal barriers they faced, and the time, effort, and money they had to put into overcoming these. Legal challenges varied between countries, as expected; one father faced more difficulties than most, explaining: ‘I had to sue her [the surrogate] in court for custody because she still had official custody, that’s how it works in [place]’. Another father described the stress he experienced when legal issues arose during the pregnancy, describing the journey as ‘a long one, it was tough’. Another father similarly experienced strain over the number of legal processes involved throughout, describing: ‘I had to do the parent order, which was stressful as well’. Other external circumstances outside of the fathers’ control included complications in the pregnancy, such as difficulties with the surrogate becoming pregnant, and challenges arising from the Covid-19 pandemic. In addition, challenges were also described by a few of the fathers in terms of their lack of adequate support. Whilst many fathers found and gathered sufficient resources and help, this was not true of all the fathers: ‘I didn’t really know any guys who had babies … they weren’t like my age, they were guys in like their 50’s and guys who were a lot older, so there wasn’t really that many people, and there also wasn’t that many support groups about surrogacy’. Likewise, another father explained that he felt he ‘never found an equal that was going through this process’ or found in terms of groups there were ‘not many, honestly, that were very helpful’.

###### Gatekeepers

While many of the fathers reported positive interactions with agencies and clinics, and often described them as helpful facilitators of the process, some fathers had more difficult experiences. One father had a challenging time with a specific member of staff at an agency, recalling: ‘some issues with the person that was matching us, she tried to force me into a surrogate’. A minority of fathers also explained that they did not have a choice about the surrogate—the process was done by the clinic and the surrogate: ‘they just said … here’s [name] … she’s going to be your surrogate, yeah. Though she got to read my profile and then agreed to be my surrogate’. Occasionally, the fees for the agency were reported as ‘not very transparent’, and fathers described feeling that they had no choice to pay the high agency fees in order to have a child.

###### Location, location, location

Being far away from the surrogate often led to some worries for the fathers. For some fathers, the location they chose to have surrogacy was felt to have had a significant impact on their experience. One father described how he had imagined it to be more typical to form a close relationship with the surrogate in another context, yet for him, he felt the location he had chosen, and the way surrogacy was typically undertaken in that country, ended up in the surrogate not wanting to form a close relationship, resulting in him feeling a ‘bit shocked, I wasn’t ready for it’. For other fathers, they decided to have a surrogacy arrangement closer to home yet faced greater costs as a result. One father justified his choice by explaining: ‘It’s also a country I’m fairly familiar with … even though it was more expensive, it just felt as a bit more … one where I could navigate more easily’.

###### Complexities with the surrogate relationship

A few of the fathers described hurdles in their relationship with the surrogate, either during or after the pregnancy. Some of the fathers who had used compensated surrogacy recognized the complexities of the relationship: ‘I think that there’s still a financial transaction in it, there’s still a sort of power in balancing that … even though I haven’t got a lot of financial resources, I’ve got more financial resources than she does’.

For other fathers, complexities sometimes emerged because of differing desires for contact and closeness. At times, expectations of being involved in the key milestones of a pregnancy, such as hospital scans, did not play out. For example, one father described how it was the surrogate’s third pregnancy ‘so it wasn’t a big deal, so she didn’t engage me in that [the scan] and so I missed that’. Similarly, another father wanted to be in more frequent contact with the surrogate, and explained how ‘it was a matter of just pointing out look, sorry, while both of our expectations are now polar apart we’re going to have to somehow compromise’. In contrast, some of the fathers found that they were comfortable with not being as closely involved as they had initially imagined; one father recalled: ‘after one or two scans I was like I didn’t even know what I was looking at … I realised I wasn’t as emotional as I kind of should be so … yeah, I didn’t really go to all the scans’. Others commented on how they were less close with the surrogate than they expected they would be: ‘She has … sort of gradually distanced herself as time has gone by’, which the father had not expected, and wished they were more in touch.

##### Theme three: Special relationship

The third theme reflects the finding that despite not one uniform description of their relationship with the surrogate, most of the fathers reported a positive relationship with the surrogate, which was conceptualized in several key ways, representing a special relationship.

###### Gratitude and amazement

Across the sample, gratitude was often expressed towards the surrogate. One father expressed: ‘She was really generous in what she did … I’ve only got love and admiration for her’. Another father said: ‘I could never thank her enough for what she’s done, I just love my life and I love having him in it’. Similarly, one father described the surrogate as having given him ‘the gift of [child’s] life’.

###### Helping to feel involved

For many of the fathers who expressed a desire to be actively involved throughout the pregnancy, the surrogates found ways to help facilitate this. One father explained how he and the surrogate were ‘in contact every day until [child] was born’ and another described that ‘we [father and baby] saw her every couple of days’ for the month after the birth. Some fathers said that the surrogate kept in close contact, enabling the fathers to feel connected to the pregnancy: ‘During that whole period though we were doing FaceTime, texting daily, all that type of stuff. Also going through the different trimesters going to physically be there and see them and go through that whole process with them’. One father reflected on how he appreciated that he was able to stay in touch with his surrogate throughout the journey: ‘I think a lot of men that I see going through this I think are very naïve about the process, it was important for me to feel connected to it [the process]. I think if I had have turned up and just was presented with a newborn child it was shocking in itself having to adjust to all of that, I think it would have made it even more challenging in that first sort of phase’.

###### Friendship

In some cases, fathers described the relationship they had developed with the surrogate as particularly close: ‘She was such an amazing woman, she was so supportive’. Similarly, another father explained: ‘So [surrogate], I’m very close to and, you know, we’ve really bonded’. One father not only reported having a good relationship with the surrogate, but also her husband: ‘we were very good friends and we laughed and joked’, describing how he ‘knew that the match was perfect’.

###### Family-like

Some fathers’ descriptions of their relationship with the surrogate included references to familial roles or bonds. Some of the fathers used ‘extended family’ to explain their gratitude towards the surrogate alongside the fact that in other circumstances, they would not necessarily have clicked or had characteristics in common to foster a friendship: ‘I want to keep in touch with her, but it’s like a cousin, I’m going to see her from time to time, but it’s … and we have a good relationship, but we wouldn’t be friends probably’. Other fathers labelled her as ‘the aunty’ or ‘the god fairy’, and one father explained: ‘she is the godmother for [child]’. Similarly, another described their relationship as ‘like extended family members’, while another father wished that he lived closer to the surrogate: ‘I think they’d be a real part of my family’.

## Discussion

This study provides a valuable insight into single fathers’ experiences of using egg donation and surrogacy to start a family, offering three key reflections. Firstly, using egg donation and surrogacy enabled single fathers to become parents in a novel way that was previously inaccessible, and allowed them to make choices and decisions about aspects of the process that were important to them. Secondly, challenges and constraints were still encountered, including the process not being entirely transparent, the necessity of international surrogacy arrangements, and experiences not matching expectations. Thirdly, despite the challenges, the fathers mainly had positive relationships with the surrogate and continued to be in contact with her. Overall, fathers’ narratives show that becoming a parent through surrogacy is a nuanced and complex experience.

The present study sheds light on the decisions and choices made by single fathers as they navigate this path to parenthood. In terms of the most desirable characteristics when choosing the egg donor, good health, and general appearance were seen by most of the fathers as the two important criteria, reflecting the choices of same-gender male couples when choosing an egg donor (Teschlade, 2018). The findings revealed that an identifiable donor was a more popular choice among the fathers than using an anonymous donor; 13 of the 21 fathers had used an identifiable donor. It is important to remember that for some fathers, having an identifiable donor meant that the fathers already knew the identity of the donor, and in some cases had spoken with their donors prior to treatment. The different categories of donation are increasingly being blurred (Jadva *et al.*, 2018). Further to this, all those who had used an identifiable donor reported that this was their preference, highlighting the emphasis the fathers put on their child being able to access information about the egg donor in the future, whereas out of the eight fathers who had used an anonymous donor, two felt that they would have preferred the donor to be identifiable, and two had been open to using either an anonymous or identifiable egg donor. It is important to note that, among the few fathers that preferred an identifiable donor but had instead used an anonymous donor, their accounts included some references to the possibility of them piecing together information to find out the donor’s identity. Firstly, this points to the growing phenomenon of more people accessing information about genetic relatives, including donors, through the increased accessibility and affordability of direct-to-consumer genetic testing (Harper *et al.*, 2016), which can result in donor-conceived people finding out information about their donor outside of ‘official’ routes, such as via clinics. Secondly, this suggests that, at the point of choosing a donor, intended parents need to be provided with comprehensive, accessible information, by agencies and clinics, to help all parents make informed decisions, and to assist parents to better understand the implications of different types of gamete donation. Research by Lysons *et al.* (2022) revealed that parents of 5-year-old children who used identifiable donation sometimes did not show a full understanding of the type of egg donation they had used. The present research further emphasizes the importance of enabling parents to be fully informed in their decisions when using donated gametes. In the longer term, this would hopefully lessen the number of parents who are not fully satisfied with the choices they make around donor identifiability/anonymity.

One of the key findings was that the fathers were able to make choices and decisions that reflected their preferences, hence had the ability to shape their journey to parenthood and establish networks to support them. The emphasis on ‘choice’ parallels Murphy’s (2013) study of coupled gay fathers whose strong desire and determination to become a parent despite significant barriers reversed out-dated narratives about the (im)possibilities of gay men becoming parents. Regarding the specific choices they made, the fathers in the present study described how pursuing surrogacy was important because of the significance of genetic relatedness to them. This was firstly expressed through a preference for gestational surrogacy as they preferred for the surrogate to not be genetically related to the child. That the fathers did not want the surrogate’s egg to be used suggests that they may have felt their relationship with their child could have been threatened if the carrier of their child was also genetically related to them, reflecting the same concerns held by same-gender male couples who used surrogacy (Blake *et al.*, 2017). The fathers’ concerns across these two samples could be interpreted in light of both the social emphasis on motherhood (Hays, 1996) and the cultural emphasis on genetic familial connections (Nordqvist, 2017), alongside the legal context, whereby using the surrogate’s egg adds additional layers of complexity. In addition, the fathers’ emphasis on genetic connections highlights the symbolic nature of being a genetic parent, as discussed by Murphy (2013), and the prevalence of long-standing discourse on what defines ‘parenthood’ despite the fathers taking what might be termed as an ‘non-traditional’ route to this life event.

That some of the fathers felt that they would have a lower priority for an adoption placement than coupled or heterosexual applicants relates to research on stigma within the adoption process. For example, a UK study that analysed social work assessments found that gay men wishing to become an adoptive or foster parent sometimes faced discriminatory and homophobic attitudes (Hicks, 2005). Seen through an intersectional lens (Collins, 1998; Bowleg, 2008), the present findings reveal how various aspects of fathers’ identities, such as relationship status and gender, appeared to have an additive effect in fathers’ perceptions of others’ views of their ability to become an adoptive parent, mirroring Boyer’s (2007) finding that same-gender male couples experienced ‘double stigma’ when adopting. Further, the findings indicate that despite a large body of research demonstrating that family structure is less influential than the quality of family relationships for children’s psychological adjustment (Golombok, 2020), single fathers still experience prejudice (Carone *et al.*, 2021a). Therefore, being able to access surrogacy offers an important family building option for single men who are pursuing a long-awaited desire to become a father.

This study is among the first to consider single fathers’ experiences of navigating a surrogacy arrangement and their relationship with the surrogate. The findings revealed that the fathers’ experiences of the surrogacy journey and relationship with the surrogate are heterogenous. Yet, overall, the fathers’ experiences largely reflected those reported by coupled parents who used surrogacy to start a family. As with two-father families (Blake *et al.*, 2016; Carone *et al.*, 2018), the fathers often maintained a relationship with the surrogate. In the present study, the qualitative content analysis and thematic analysis demonstrated that many of the fathers described a positive relationship with the surrogate, with the remaining either having a neutral or ambivalent relationship, and about two thirds of the sample were in touch with the surrogate at least every 3 months. This replicates Jadva *et al.*’s (2019) finding that gay and heterosexual couples mostly had a very positive or positive relationship with their surrogate. In addition, the findings across the two studies are similar in that many parents described the surrogate as part of their extended family or as a friend. The findings also reflect those of two other studies. Firstly, a study of 34 surrogates in the UK (Imrie and Jadva, 2014), reported that most of the surrogates were still in touch with the heterosexual couples they had been the surrogate for 5–15 years later, and generally had positive relationships with the couples. Secondly, research on heterosexual couples who had used surrogacy to form a family (Jadva *et al.*, 2012) found that 60% of the parents were still in touch with the surrogate 10 years after the birth of their child. Further, the findings regarding the fathers’ thoughts and feelings about the surrogate suggest that they viewed the surrogate as having a special role in their lives. The gratitude expressed by many of the fathers towards the surrogate mirrors the feelings of children of single fathers when discussing their thoughts and feelings about having been conceived via surrogacy (Carone *et al.*, 2018), and also reflects the ways in which single mothers have been found to describe sperm donors, for example, as a ‘gift-giver’ (Zadeh *et al.*, 2016).

Although positive in many ways, it is important to note that the findings of this study also highlight the challenges that are faced by intended parents as they navigate surrogacy. Previous research has shown that surrogacy arrangements can be difficult; pregnancies can come with complications, and that the practice of surrogacy varies from country to country (Jadva *et al.*, 2019). In line with research on couples who use transnational surrogacy (Riggs *et al.*, 2015; Ziv and Freund-Eschar, 2015; Carone *et al.*, 2017; Jadva *et al.*, 2019), the more difficult aspects of surrogacy reported by the fathers in the present study included: wanting more of a say in the decisions made about the surrogacy arrangement by clinics or agencies, needing more support, or feeling less involved in the pregnancy than they would have liked, often due to geographical distance. Also relating to a lack of choice over aspects of the surrogacy process, some fathers described having faced legal difficulties in obtaining a parental order. Such findings echo prior research on the legal challenges faced by men who pursue surrogacy to become parents (Arvidsson *et al.*, 2019). For the present sample, a more detailed exploration of the legal complexities faced by solo fathers are explored in Zadeh *et al.* (2022). The current findings, together with previous research, highlight the legal challenges faced by single men who use surrogacy regardless of whether surrogacy is legal or not in the country they reside in.

Regarding its theoretical contributions, this study highlights the usefulness of studying the intersections between different aspects of fathers’ identities (Collins, 1998; Bowleg, 2008). In the present investigation, fathers’ identities intersected in ways that on the one hand created barriers and additional challenges (especially legal) and on the other hand enabled choice and autonomy. Paralleling Berkowitz’s (2020) reflection that gay men who become fathers through surrogacy experience both privilege and marginalization, among the present sample, fathers simultaneously benefitted from the aspects of their identity and experiences that enabled them to have the resources and opportunity to pursue surrogacy, yet at the same time, they faced legal and practical barriers to becoming a parent as well as feelings of isolation during the surrogacy journey.

This article reported on the accounts of single men who used surrogacy and egg donation to start a family. The findings of this study are based on a small international sample of single fathers who had used surrogacy, and therefore tell us little about how specific regulations and rules in different countries relate to single men’s experiences of surrogacy, or about changes in the relationship between a single father and the surrogate over time, as the children grow older and start to understand about their conception. Yet, considering there is little research on single men who seek to become parents through surrogacy, the present study increases understanding of the experiences of single men who pursue this family-building option. Whilst many of the fathers’ experiences reflected those of two-father families pursuing surrogacy, there were also unique experiences associated with single parenthood.

The findings point to the importance of social support, and the provision of resources tailored to the needs of single fathers. Attention to these issues will be of benefit to single men who choose to start a family through surrogacy. In terms of practical implications, effective counselling is important for intended parents and surrogates (van Den Akker, 1998). For single men pursuing parenthood, counselling can offer a point of reflection to think about wants and expectations regarding contact around and after the birth, and what the father intends to disclose to his child about his route to parenthood. However, the type and accessibility of counselling during the process of assisted reproduction varies between, and within, countries (Blyth, 2012), and needs to be adequately tailored for different types of intended parent(s). The opportunity for intended parents to have access to multiple counselling sessions should be provisioned by agencies and clinics, who act as both facilitators and gatekeepers of surrogacy for single men. Alongside being transparent about the process, providing information on other parents’ experiences, especially regarding single fathers, may also be beneficial. As it is hard for single men to access surrogacy in many countries, international surrogacy arrangements, which can come with more challenges, are more likely. As previously asserted by Ziv and Freund-Eschar (2015), given the disparities in how surrogacy is practiced globally, there is a need for robust international guidelines to help support intended parents seeking international surrogacy.

Given that surrogacy is now more accessible to single men, there is a notable lack of research focus on this family type. Future research should explore the role of egg donors in the lives of single father families over time given the importance placed by many single men upon knowing the identity of the donor. There is also a need for more inclusive samples of single father families that reflect a range of different aspects of identity, such as ethnic identity and socio-economic status. Given the diverse ways in which surrogacy is now practiced (Jadva, 2020), understanding the impact on the families created will become even more important, particularly as the number of single men using surrogacy for family building steadily increases.

## Data Availability

The data underlying this article cannot be shared publicly to protect the privacy of individuals that participated in the study.

## References

[dead152-B1] Arvidsson A , Johnsdotter S, Emmelin M, Essén B. Being questioned as parents: an interview study with Swedish commissioning parents using transnational surrogacy. Reprod Biomed Soc Online 2019;8:23–31. 30911689 10.1016/j.rbms.2018.08.001 PMC6416408

[dead152-B2] Berkowitz D. Gay men and surrogacy. In: Goldberg AE, Allen KR (eds). LGBT-Parent Families. Innovations in Research and Implications for Practice. New York: Springer, 2020, 43–160.

[dead152-B3] Birenbaum-Carmeli D , Montebruno P. Incidence of surrogacy in the USA and Israel and implications on women's health: a quantitative comparison. J Assist Reprod Genet 2019;36:2459–2469. 31673853 10.1007/s10815-019-01612-9 PMC6911123

[dead152-B4] Blake L , Carone N, Slutsky J, Raffanello E, Ehrhardt AA, Golombok S. Gay father surrogacy families: relationships with surrogates and egg donors and parental disclosure of children's origins. Fertil Steril 2016;106:1503–1509. 27565261 10.1016/j.fertnstert.2016.08.013 PMC5090043

[dead152-B5] Blake L , Carone N, Raffanello E, Slutsky J, Ehrhardt AA, Golombok S. Gay fathers’ motivations for and feelings about surrogacy as a path to parenthood. Hum Reprod 2017;32:860–867. 28333218 10.1093/humrep/dex026 PMC5400050

[dead152-B6] Blyth E. Guidelines for infertility counselling in different countries: is there an emerging trend? Hum Reprod 2012;27:2046–2057. 22493028 10.1093/humrep/des112

[dead152-B7] Bowleg L. When Black + lesbian + woman ≠ Black lesbian woman: the methodological challenges of qualitative and quantitative intersectionality research. Sex Roles 2008;59:312–325.

[dead152-B8] Boyer CA. Double stigma: the impact of adoption issues on lesbian and gay adoptive parents. In: Javier RA, Baden AL, Biafora FA, Camacho-Gingerich A (eds). Handbook of Adoption: Implications for Researchers, Practitioners, and Families. California: SAGE Publications, 2007, 228–241.

[dead152-B9] Braun V , Clarke V. Using thematic analysis in psychology. Qual Res Psychol 2006;3:77–101.

[dead152-B10] Braun V , Clarke V. Reflecting on reflexive thematic analysis. Qual Res Sport Exerc Health 2019;11:589–597.

[dead152-B11] Braun V , Clarke V. Thematic Analysis: A Practical Guide. California: SAGE Publications, 2021.

[dead152-B12] Carone N , Baiocco R, Lingiardi V. Single fathers by choice using surrogacy: why men decide to have a child as a single parent. Hum Reprod 2017;32:1871–1879. 28854718 10.1093/humrep/dex245

[dead152-B13] Carone N , Baiocco R, Lingiardi V, Barone L. Gay and heterosexual single father families created by surrogacy: father–child relationships, parenting quality, and children’s psychological adjustment. Sex Res Soc Policy 2020;17:711–728.

[dead152-B14] Carone N , Baiocco R, Manzi D, Antoniucci C, Caricato V, Pagliarulo E, Lingiardi V. Surrogacy families headed by gay men: relationships with surrogates and egg donors, fathers' decisions over disclosure and children's views on their surrogacy origins. Hum Reprod 2018;33:248–257. 29237004 10.1093/humrep/dex362

[dead152-B15] Carone N , Barone L, Lingiardi V, Baiocco R, Brodzinsky D. Factors associated with behavioral adjustment among school-age children of gay and heterosexual single fathers through surrogacy. Dev Psychol2021a;57:535–547. 33661670 10.1037/dev0001155

[dead152-B16] Carone N , Manzi D, Barone L, Lingiardi V, Baiocco R, Bos HMW. Father–child bonding and mental health in gay fathers using cross-border surrogacy during the COVID-19 pandemic. Reprod Biomed Online 2021;43:756–764. 34417139 10.1016/j.rbmo.2021.05.023 PMC8819845

[dead152-B17] Coles RL. Single-father families: a review of the literature. J Fam Theory Rev 2015;7:144–166.

[dead152-B18] Collins PH. It’s all in the family: intersections of gender, race, and nation. Hypatia 1998;13:62–82.

[dead152-B19] Department of Health and Social Care. *The surrogacy pathway: surrogacy and the legal process for intended parents and surrogates in England and Wales*, 2021, July 23. https://www.gov.uk/government/publications/having-a-child-through-surrogacy/the-surrogacy-pathway-surrogacy-and-the-legal-process-for-intended-parents-and-surrogates-in-england-and-wales

[dead152-B20] Flick U. An Introduction to Qualitative Research, 5th edn. California: SAGE Publications, 2014.

[dead152-B21] Gair S. Feeling their stories: contemplating empathy, insider/outsider positionings, and enriching qualitative research. Qual Health Res 2012;22:134–143. 21873286 10.1177/1049732311420580

[dead152-B22] Golombok S. Modern Families: Parents and Children in New Family Forms. Cambridge: Cambridge University Press, 2015.

[dead152-B23] Golombok S. We Are Family: What Really Matters for Parents and Children. London: Scribe, 2020.

[dead152-B24] Golombok S , Blake L, Slutsky J, Raffanello E, Roman G, Ehrhardt A. Parenting and the adjustment of children born to gay fathers through surrogacy. Child Dev 2018;89:1223–1233. 28111745 10.1111/cdev.12728 PMC6055684

[dead152-B25] Golombok S , Zadeh S, Imrie S, Smith V, Freeman T. Single mothers by choice: mother-child relationships and children's psychological adjustment. J Fam Psychol 2016;30:409–418. 26866836 10.1037/fam0000188 PMC4886836

[dead152-B26] Gregory A , Milner S. What is ‘new’ about fatherhood? The social construction of fatherhood in France and the UK. Men Masc 2011;14:588–606.

[dead152-B27] Harper JC , Kennett D, Reisel D. The end of donor anonymity: how genetic testing is likely to drive anonymous gamete donation out of business. Hum Reprod 2016;31:1135–1140. 27073260 10.1093/humrep/dew065

[dead152-B28] Hays S. The Cultural Contradictions of Motherhood. London: Yale University Press, 1996.

[dead152-B2900] Hicks S . Lesbian and gay foster care and adoption: A brief UK history. Adopt Foster 2015;29:42–56.

[dead152-B29] Imrie S , Golombok S. Impact of new family forms on parenting and child development. Annu Rev Dev Psychol 2020;2:295–316.

[dead152-B30] Imrie S , Jadva V. The long-term experiences of surrogates: relationships and contact with surrogacy families in genetic and gestational surrogacy arrangements. Reprod Biomed Online 2014;29:424–435. 25131555 10.1016/j.rbmo.2014.06.004

[dead152-B31] Jadva V. Postdelivery adjustment of gestational carriers, intended parents, and their children. Fertil Steril 2020;113:903–907. 32312560 10.1016/j.fertnstert.2020.03.010

[dead152-B32] Jadva V , Badger S, Morrissette M, Golombok S. “Mom by choice, single by life’s circumstance … ”: findings from a large scale survey of the experiences of single mothers by choice. Hum Fertil (Camb) 2009;12:175–184. 19895361 10.3109/14647270903373867

[dead152-B33] Jadva V , Blake L, Casey P, Golombok S. Surrogacy families 10 years on: relationship with the surrogate, decisions over disclosure and children's understanding of their surrogacy origins. Hum Reprod 2012;27:3008–3014. 22814484 10.1093/humrep/des273 PMC3442632

[dead152-B34] Jadva V , Freeman T, Tranfield E, Golombok S. Why search for a sperm donor online? The experiences of women searching for and contacting sperm donors on the internet. Hum Fertil (Camb) 2018;21:112–119. 28449623 10.1080/14647273.2017.1315460 PMC5951149

[dead152-B35] Jadva V , Gamble N, Prosser H, Imrie S. Parents' relationship with their surrogate in cross-border and domestic surrogacy arrangements: comparisons by sexual orientation and location. Fertil Steril 2019;111:562–570. 30827525 10.1016/j.fertnstert.2018.11.029 PMC6408321

[dead152-B36] Jadva V , Murray C, Lycett E, MacCallum F, Golombok S. Surrogacy: the experiences of surrogate mothers. Hum Reprod 2003;18:2196–2204. 14507844 10.1093/humrep/deg397

[dead152-B37] Jadva V , Prosser H, Gamble N. Cross-border and domestic surrogacy in the UK context: an exploration of practical and legal decision-making. Hum Fertil (Camb) 2021;24:93–104. 30537445 10.1080/14647273.2018.1540801

[dead152-B38] Johnson KM. Single, straight, wants kids: media framing of single, heterosexual fatherhood via assisted reproduction. J Gender Stud 2017;26:387–401.

[dead152-B39] Jones C , Zadeh S, Jadva V, Golombok S. Solo fathers and mothers: an exploration of well-being, social support and social approval. IJERPH 2022;19:9236. 35954593 10.3390/ijerph19159236 PMC9368669

[dead152-B40] Lysons S , Imrie S, Jadva V, Golombok S. ‘I’m the only mum she knows’: parents’ understanding of, and feelings about, identity-release egg donation. Hum Reprod 2022;37:2426–2437. 36006027 10.1093/humrep/deac174 PMC9527462

[dead152-B41] Mayring P. Qualitative Content Analysis: Theoretical Background and Procedures. New York: Springer, 2015, 365–380.

[dead152-B42] McCorkel JA , Myers K. What difference does difference make? Position and privilege in the field. Qual Sociol 2003;26:199–231.

[dead152-B43] Murphy DA. The desire for parenthood: gay men choosing to become parents through surrogacy. J Fam Issues 2013;34:1104–1124.

[dead152-B44] Neergaard MA , Olesen F, Andersen RS, Sondergaard J. Qualitative description – the poor cousin of health research? BMC Med Res Methodol 2009;9:52. 19607668 10.1186/1471-2288-9-52 PMC2717117

[dead152-B45] Nordqvist P. Genetic thinking and everyday living: on family practices and family imaginaries. Sociol Rev 2017;65:865–881.

[dead152-B4500] Riggs DW , Due C, Power J. Gay men‘s experiences of surrogacy clinics in India. J Fam Plann Reprod Health Care 2015;41:48–53. 25351689 10.1136/jfprhc-2013-100671

[dead152-B46] Shenkman G , Levy S, Winkler ZB-D, Bass D, Geller S. Higher levels of postnatal depressive symptomatology, post-traumatic growth, and life satisfaction among gay fathers through surrogacy in comparison to heterosexual fathers: a study in Israel in times of COVID-19. IJERPH 2022;19:7946. 35805604 10.3390/ijerph19137946 PMC9265351

[dead152-B47] Teschlade J. *Conceiving before conception: gay couples searching for an egg donor on their journey to parenthood* . In: Mitra S, Schicktanz S, Patel T (eds). Cross-Cultural Comparisons on Surrogacy and Egg Donation. London: Palgrave Macmillan, 2018, 301–323.

[dead152-B48] Tsfati M , Segal-Engelchin D. The social experiences of single gay fathers in Israel: an intersectional perspective. IJERPH 2022;19:11356. 36141626 10.3390/ijerph191811356 PMC9517099

[dead152-B49] van Den Akker OB. Functions and responsibilities of organizations dealing with surrogate motherhood in the UK. Hum Fertil (Camb) 1998;1:10–13. 11660783

[dead152-B50] Wall G , Arnold S. How involved is involved fathering? An exploration of the contemporary culture of fatherhood. Gender Soc 2007;21:508–527.

[dead152-B51] Zadeh S , Freeman T, Golombok S. Absence or presence? Complexities in the donor narratives of single mothers using sperm donation. Hum Reprod 2016;31:117–124. Doi: 10.1093/humrep/dev275. 26545622 PMC4677963

[dead152-B52] Zadeh S , Jadva V, Golombok S. Documenting families: paper-work in family display among planned single father families. Sociology 2022;56:859–875. 10.1177/00380385211073238 PMC7618298 41141418

[dead152-B53] Ziv I , Freund-Eschar Y. The pregnancy experience of gay couples expecting a child through overseas surrogacy. Fam J 2015;23:158–166.

